# In vitro characterisation of Bovine Leukemia Virus capsid protein self-assembly

**DOI:** 10.1186/1742-4690-8-S1-A30

**Published:** 2011-06-06

**Authors:** Gonzalo Obal, Jean Lepault, Federico Carrion, Lorena Tome, Gonzalo Moratorio, Gonzalo Rama, Sergio Bianchi, Otto Pritsch

**Affiliations:** 1 Unidad de Biofísica de Proteínas, Institut Pasteur de Montevideo, Montevideo, 11400, Uruguay; 2 CNRS, Laboratoire de Virologie Moléculaire et Structurale, Gif-sur-Yvette, 91198, France

## 

Bovine leukemia virus (BLV) and Human T-cell Leukemia virus (HTLV) are oncogenic retroviruses of the genus Deltaretrovirus, and affects cattle and human respectively. BLV is the etiologic agent of Enzootic Bovine Leukemia and infects B-cells of dairy/beef cattle generating a life-long infection leading to economic losses and commercial restraints. In common with other retroviruses, formation of a functional core structure during morphogenesis of BLV/HTLV viral particles is essential for infectivity. In this process, thousands of capsid (CA) molecules self-assemble to form a shell which encases the genome. The assembly mechanism of BLV-CA protein assemble to form mature-type core in vivo and in vitro are unknown, and more broadly, they are yet poorly understood in Deltaretrovirus, despite important advances utilizing HTLV-CA protein. We recently started in vitro analysis of the characteristics/requirements of BLV-CA protein assembly and developed a turbidimetry-based assembly assay using the purified recombinant BLV-CA protein. Here we show the examination of assembly under a variety of environmental and physical, including near physiological, conditions. Specifically, the effect on self-assembly triggering and kinetics was analysed for protein concentration, pH, ionic strength, temperature, phosphate and polyphosphates. The influence of both independent N-terminal and C-terminal domains on oligomerization kinetics was also assessed. In parallel, we performed electron microscopic analysis of assembly products in order to evaluate the physical characteristics of the material formed under these conditions. This work provides the first description of the BLV capsid protein assembly properties, which may also be of relevance for understanding other Deltaretrovirus assembly characteristics.

